# A study of the impact of corporate digitization on environmental protection: Take Chinese A-share companies in Shanghai and Shenzhen as an example

**DOI:** 10.1371/journal.pone.0285896

**Published:** 2023-05-25

**Authors:** Zexia Zhao, Jun Yan

**Affiliations:** School of Finance and Economics, Jiangsu University, Zhenjiang, 212013, China; Shenzhen University, CHINA

## Abstract

Textual analysis and the Entropy-TOPSIS method are used in this research to create a measure of corporate environmental protection, and multiple regressions are used to find out how digitalization affects corporate environmental protection. The research sets up a theoretical framework for how corporate digitalization affects environmental protection and looks into how external financing constraints and an organization’s own financial position play a role in the middle. The research then looks at how outside factors like the business environment of the market and the level of competition in the industry affect the relationship. Using a threshold regression approach, the research also examines the change in the impact of digitalization on environmental protection after investor sentiment crosses the threshold from the distinct perspective of investor sentiment. Our research provides theoretical support for environmental protection by corporations and government policy direction.

## 1 Introduction

As human society has become more productive, it has created a lot of material and spiritual wealth. Nevertheless, productive activities inexorably consume resources and impose a certain burden on ecological cycles [1]. The Earth has limited resources, and nature’s ability to clean up pollution is slow and limited [2]. When human production develops at a rate that exceeds what nature can support, severe environmental pollution problems result [3]. Currently, humans expand production blindly and exploit limited resources irresponsibly, causing ecological damage that is difficult to restore in the short term. According to the 2018 Industrial Development Report by the United Nations Industrial Development Organization, thriving industrial development requires a sustainable environment. Due to industrial shifts and industry barriers, the most polluting firms are typically concentrated in developing nations [4]. What is the state of environmental pollution and administration in China, the world’s most populous developing nation? The Bulletin on the Condition of China’s Ecological Environment shows that China’s management of the environment could be better in many ways. In 2019, the air quality in more than half of China’s cities is still below standards. Large freshwater lakes like Taihu Lake are still mildly polluted. Soil erosion is still happening over an area of 2,736,900 square kilometers. In 2019, China will invest 9,152 billion yuan in ecology, up from 5,258 billion yuan in 2009. China should establish a balance between enhancing economic vitality and promoting sustainable development. President Xi Jinping has since emphasized the importance of high-quality development in constructing a new socialist China on a number of significant occasions. In 2022, at the opening of the 20th National Congress of the Communist Party of China, Xi Jinping once again highlighted high-quality development as a top priority in constructing a socialist nation, giving it the necessary political attention. However, environmental protection has significant public attributes, and government focus alone is insufficient. As significant participants in the market economy, businesses are obligated to take environmental pollution seriously and implement effective solutions.

China is currently part of the Industry 4.0 era. Currently, information and data are used as a means of production to assist the market in functioning more efficiently. China’s State Council published "Opinions on Building a More Perfect Institutional Mechanism for the Market-based Allocation of Factors" in 2020, which proposes to highlight the role of information technology and guide the formation of a market that uses data as a factor of production, thereby injecting new life into the national economy. The digital economy refers to a set of economic activities that use modern information networks as a carrier, digitize information in the production and operation of enterprises as a factor of production, improve the efficiency and accuracy of decision-making through modern information technology, and promote economic growth by enhancing production efficiency and industrial transformation [5,6]. China’s government is at the forefront of the digital economy. The Chinese government is guiding the progressive penetration of the digital economy into social governance, business production, and people’s lives. The digital economy enables users to make more precise judgments by providing faster, cheaper access to more comprehensive data [7]. Consequently, what role can the digital economy play in corporate environmental governance?

What variables influence environmental protection? The majority of research has focused on corporate finance [8], industrial structure [9], and policy orientation [10]. A company’s solid financial position gives it the confidence to pursue endeavors that appear unrelated to its near-term profitability [11]. Sievers and Vandenberg (2007) contend that when a company has insufficient liquidity, it will make conservative decisions and abandon initiatives that contribute to long-term growth in order to ensure its survival [12]. Using a sample of Chinese high-tech SMEs, Xiang et al. (2018) discovered that with improved financial conditions, businesses are able to invest more in technological innovation to improve the quality of their development and reduce environmental contamination [13]. The industry of a company can influence the environmental pollution it causes [14]. Li et al. (2021) conducted a study from the perspective of 30 Chinese provinces and discovered that firms in high-tech industries tend to produce less environmental contamination while delivering greater economic benefits [15]. Consequently, some academics have proposed upgrading regional industrial structures when conducting regional economic studies [16]. Government policies can provide conceptual direction. After government departments advocate for environmental protection and introduce relevant laws and regulations, businesses will adapt their business strategies to avoid penalties, and regions will follow the directives of higher authorities to tighten local restrictions [17]. Zhang et al. (2021) studied a sample of mining companies in China and found that tax incentives for green development can encourage technological innovation and, consequently, environmental protection by encouraging companies [18].

Moreover, what are the consequences of the digital economy? The digital economy will bring about changes in production efficiency, industrial structure, and regulatory strategies. The digital economy employs the Internet information technology of the third industrial revolution to use information data as a means of production, thereby making the production process more transparent and enabling production departments to better control the production process [19]. After the introduction of the digital economy, Wang et al. (2022) discovered that the form of power generation and the structure of energy consumed by the power company were optimized, resulting in an increase in energy output per unit [20]. Using a sample of Chinese provinces from 2014 to 2020, Wu & Yang (2022) discovered that the development of the digital economy had an effect on China’s industrial structure [21]. As digitalization spreads, positions in certain secondary industries will be filled by intelligent machines that can be controlled by inputting programs. After a certain period of buffering, the displaced labor force will transition to occupations requiring human initiative and training, thereby contributing to the industry’s restructuring. Digitalization can also help companies better integrate rich, fragmented, and massive amounts of data [22]. And Rowbottom et al. (2021) argue that digitalization enables companies to limit their discretion in disclosing accounting information, thereby making it more compliant with accounting standards [23]. In addition, when an accounting information system is widely adopted, the information disclosed by businesses becomes more uniform and user-friendly.

How, then, does the digitalization of enterprises impact environmental protection from a macroeconomic and microbusiness perspective? There is still space for extensive research expansion in this field. This investigation begins with an introduction. It describes the history of China’s digital economy and environmental governance. In addition, we examine the pertinent literature in this section. The theoretical analysis and research design constitute the second section. We conduct a theoretical analysis of how the digitalization of businesses impacts environmental governance and develop a model based on the hypothesis. The third section is devoted to the research methodology. Here, we present the foundation for data selection and variable measurement. The fourth section contains the empirical test results. Here, the hypotheses are evaluated, and additional analysis is offered. The fifth segment is the discussion, and the sixth section concludes with an outlook based on the paper’s limitations.

The following are the minor contributions of this study: (1) We have developed an indicator to measure a company’s environmental protection efforts. We believe that a company’s environmental protection should include the significance it places on environmental protection, the actions it has taken, and the results it has achieved. This study combines text learning with the Entropy-TOPSIS method to develop an environmental protection index. (2) The commercial impact of digitalization on environmental protection was investigated. The impact of digitalization on environmental protection has received less attention in prior research. Even when scholars have focused on this aspect, they have typically analyzed it from a provincial or urban perspective. This paper examines this topic from the enterprise’s micro perspective.

## 2 Theoretical analyses and research design

In business, the digital economy refers to the digitalization of the enterprise. When a business turns digital, digital information systems can collect and organize production data more quickly, and decision support systems can assist managers in making more accurate decisions and allocating resources more efficiently. The optimization of business processes and the reorganization of production methods have given companies a new lease on life, giving them the confidence to choose initiatives with a low short-term return but long-term benefits. Environmental protection is of particular importance to businesses. However, environmental protection conflicts with the "rational economic man" character of business in terms of profitability. Funding constraints and financial difficulties are significant barriers to businesses fulfilling their responsibilities as "social men" [24]. The enterprise’s digitalization can alleviate financial issues by enhancing the efficacy of resource allocation through advanced technology and by transmitting information that facilitates financing, thereby contributing to the enterprise’s environmental protection.

### 2.1 Digitalization of enterprises directly contributes to environmental protection

The new growth theory focuses on forces within the economic system and says that these internal factors are what cause economic growth [25]. Businesses are becoming more digital, which is part of using knowledge and technology in a way that can help the economy grow. Digital technology can effectively collect and process pertinent information and data to improve productivity by optimizing the production chain [26], improving the supply chain [27], and reducing information transmission losses [28]. The increase in output per unit will inevitably lead to a decrease in the quantity of energy consumed by the company, which will have an effect on environmental pollution. This reduces environmental pollution in terms of energy consumption and production requirements. In addition to this, the knowledge spillover effect also plays a role. Digitalization can not only improve a company’s production operations, but it can also contribute to the digitalization of nearby businesses [29]. After observing other businesses obtaining advantages in production operations, for instance, as a result of digitalization, these businesses begin to recognize its value [30]. This expedites the digital transformation of the involved companies. By enabling businesses to join digitalization to construct a broader and more extensive digital platform [31], this can be more effective in enhancing business efficiency and promoting environmental protection.

Under the consensus theory, humans and nature are required to coexist in harmony. The economy, society, and environment are all interconnected, and alterations to one factor will have an impact on the others. Without the support of the environment, the orderly development of the economy and society is impossible [32]. Digitalization as a new production technology will inevitably have an effect on environmental protection. In addition, the digitalization of businesses can assist them in establishing a broader and more convenient communication platform, allowing for more effective communication and the implementation of customer demands. As humans inhabit the natural environment, environmental protection is vital to the sustainable development of human civilization and the wellbeing of individuals [33]. The ’economic’ character of business, however, has always caused companies to overlook their ’social’ nature. However, digitalization expands the opportunities for constituents to communicate with businesses and urge them to protect the environment. In addition, the legitimacy theory asserts that the implementation of environmental protection is a priority for businesses, following the introduction of government regulations and public perceptions. The accelerated process of digitalization also facilitates government surveillance.

### 2.2 Digitalization indirectly contributes to environmental protection

Ultimately, companies are profit-driven. Environmental protection necessitates production method modifications and technological advancement. These changes have a significant impact on the business and necessitate substantial capital expenditures, which are a barrier to environmental protection [34]. The enterprise’s digitalization can mitigate and enhance this issue to some extent. First, from an expense standpoint. Digitalization can assist businesses in acquiring sufficient information data and utilizing more advanced decision-making technology, thereby reducing the losses caused by poor decisions [35]. It also reduces resource loss in the production chain by easing transitions between processes. In addition, the efficiency of stakeholder monitoring of the business can be increased if digitalization is utilized effectively. After setting up the company’s workflow in strict accordance with the relevant guidelines, the digitalization-enabled information work system is able to maintain a record of everything that has been done, thereby enhancing the company’s monitoring [36]. This reduces the company’s financial burden by decreasing agency fees. Moreover, the development of the digital economy has become a trend in China’s future development, given the government’s "Digital China" and "Smart Society" initiatives. This is an opportune moment for businesses to actively engage with national policies in order to foster a positive relationship between the government and business and to instill confidence in the capital market, thereby reducing the level of financing constraints [37]. In this way, the company’s digitalization can guarantee the smooth implementation of its environmental protection projects by reducing funding restrictions.

In conjunction with the above theoretical analysis, this study presents the following hypotheses.

H1: Other things being equal, the digitalization of enterprises can contribute to environmental protection.
H2a: Other things being equal, the digitalization of companies promotes environmental protection by improving their internal financial situation.
H2: Other things being equal, the digitalization of the firm promotes environmental protection by way of the degree of external financing constraints.

**Fig 1:**
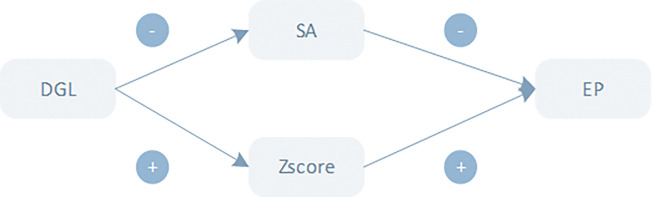
Theoretical analysis.

We have drawn a flow chart based on the theoretical analysis above, which the reader can see in Fig 1.

## 3 Research methodology

### 3.1 Data selection

This study’s sample is made up of Chinese A-shares from Shanghai and Shenzhen from 2008 to 2019. Since the end of 2007, China has had a new accounting standard that requires Chinese companies with shares on the stock market to disclose accounting information in a standard way. Late in 2019, COVID-19 broke out in China, which has caused a lot of economic and social trouble. Under this scenario, we believe that the financial data of companies may become skewed. We therefore use 2019 as the study’s cutoff year. Moreover, the selected data for this investigation has been processed as follows: (1) Excluding companies with two consecutive years of losses (with "ST" in the stock abbreviation) and companies whose listing has been suspended for the purpose of special transfers ("PT" in the abbreviation). In the event of delisting or transfer, these companies are likely to have anomalous financial data, which may influence the empirical results. (2) Excluding financial and insurance companies. Their industry codes are J66, J67, J68 and J69. Which have unique production and operation models and financing access compared to other industries. (3) Exclude samples with absent or discontinuous data. (4) Exclude businesses that have been publicly traded for less than a year. (5) To avoid the effect of extreme values, we winsorize the data by the first and last 1%. Following the preceding operations, we obtained 3,757 companies and 31,315 data items in total.

The majority of the data for this research came from the China Economic and Financial Research Database (CSMAR). Using Python for textual analysis, the section involving word frequency statistics was examined. In addition, Excel 2021 and Stata 17 were employed for data processing and analysis.

### 3.2 Variable definitions

We have listed the main variables used in this study in Table 1.

**Table 1:** Main variables.

	Symbol	Name	Interpretation/Measurement
predicted variable	EP	environmental protection	measured using the entropy method
explanatory variable	DGL	digitalization of the enterprise	proportion of expenditure on intangible assets spent on digitization of the business
mediators	SA	level of corporate finance constraints	degree of financing constraints, with higher values indicating a greater degree of financing constraints on the firm
	Zscore	financial situation	financial situation of the business, with higher values indicating a better financial position
moderator variables	markt	business environment	local business environment index, the higher the index the better the locality is for doing business
	HHI	Herfindahl-Hirschman Index	the higher the index, the more competitive the industry
controlled variables	lev	balance sheet ratio	liabilities/assets
	cash	cash situation	cash generated from current operations of the business/assets
	cur	current ratio	current assets/current liabilities
	growth	development capacity	chain ratio of main operating income
	tan	proportion of tangible assets	carrying value of tangible assets/total assets
	ind	board independence	percentage of independent directors on the board
	isp	institutional shareholding	shareholding of institutional investors as a percentage of the total number of shares of the enterprise outstanding

#### 3.2.1 Predicted variable

Formal pairs of public reports, like an organization’s annual report, social responsibility report, and environmental report, are important ways for the organization and its outside stakeholders to talk to each other. These reports not only reflect the business status of the enterprise, but they can also communicate its strategic direction [38]. This study uses a new way to combine text analysis and Entropy-TOPSIS to come up with an indicator for measuring how well companies protect the environment. After four main indicators were set up, including "Information Disclosure," the annual reports, social responsibility reports, and environmental reports of the companies were crawled in Python and their text was analyzed. To improve the indicators, each level 1 indicator was broken down into a number of level 2 indicators (see Table 2). The Entropy-TOPSIS method was then used to align and standardize the 29 secondary indicators, and their weights were calculated. This research used weights to come up with an indicator that shows how well the company protects the environment.

**Table 2:** Environmental protection indicator components.

Level 1 indicators	Level 2 indicators	Weight
Information Disclosure	Annual Reports of Listed Companies	0.005255
	Social Responsibility Report	0.029845
	Environmental Reports	0.089802
Environmental pollution	Wastewater discharge	0.024162
	Chemical Oxygen Demand Emissions	0.045940
	SO2 emissions	0.048867
	CO2 emissions	0.054914
	Soot and dust emissions	0.037689
	Industrial solid waste generation	0.071685
Environmental protection	Environmental philosophy	0.024666
	Environmental objectives	0.045629
	Environmental Management System	0.026630
	Environmental Education and Training	0.054415
	Special Environmental Action	0.043555
	Environmental incident response mechanism	0.037291
	Environmental honours or awards	0.044691
	The "three simultaneous" system	0.050698
Environmental protection results	Waste gas abatement treatment	0.025552
	Wastewater abatement treatment	0.025490
	Dust and fume control	0.038024
	Solid waste utilization and disposal	0.029811
	Treatment of noise, light pollution, radiation, etc.	0.038570
	Cleaner Production Implementation	0.041427
	Pollutant discharge compliance	0.000047
	Sudden environmental accidents	0.000020
	Environmental violations	0.000121
	Environmental petition cases	0.000007
	ISO14001 certified or not	0.033382
	ISO9001 certified or not	0.031819

#### 3.2.2 Explanatory variable

This study measures the degree to which businesses are digital based on the amount of money they invest in digital transformation. The feature word mapping in S1 Fig is based on Wu et al. (2021), which looked at the Government Work Report and other relevant Chinese government policy documents [39]. Using Python, this study compiles and aggregates the annual reports of Chinese A-share companies in Shanghai and Shenzhen for all years. If the notes in the enterprises’ annual financial reports matched the keywords in S1 Fig, they were categorized as the enterprises’ digital transformation funds. We determined the degree of digital transformation by tallying the intangible assets used for digital transformation over the course of one year and calculating their proportion of the enterprise’s intangible assets.

Some academics use the number of times a keyword appears as a metric because it shows how a company runs its business [40,41]. In this study, the number of times the keywords in S1 Fig appeared in the annual reports of businesses was counted. The entropy value method was then used to come up with an indicator of how digitally transformed businesses are. This variable also serves as a proxy for the original explanatory variables.

#### 3.2.3 Controlled variables

Too many omitted variables may have an effect on the residuals of the multiple regression equation, leading to endogeneity issues. We utilize the research of Liu & Liu (2022) and Zhang et al. (2022) to include control variables that reflect the financial position and management characteristics of the firm, such as the balance sheet ratio and monetary situation [34,42].

### 3.3 Model construction

To examine the impact of digitalization of enterprise on enterprises’ environmental protection, the following model is developed. If *β*_1_ is significantly positive, it indicates that digitalization of enterprise is conducive to increasing the firm’s environmental protection.


$${EP}_{i,t}=\alpha +\beta_{1}{DGL}_{i,t}+\sum {controls}_{i,t}+\sum industry+\sum year+\varepsilon_{i,t}$$ (1)

*EP*_*i*,*t*_ denotes environmental protection of firm t in period i. *DGL*_*i*,*t*_ denotes the digitalization of firm t in period i. *controls*_*i*,*t*_ denotes the control variables of firm t in period i. Please see Table 1 for the selection of control variables. And then we control for industry-fixed effects and time-fixed effects.

## 4 Empirical analyses and results

### 4.1 Descriptive statistic

The descriptive statistics for the primary variables are displayed in Table 3. Environmental protection of enterprises (EP) has a mean value of 0.6373, a median value of 0.6666, and a standard deviation of 0.0820. This suggests that there is little variation in environmental protection among China’s listed companies. The degree of digitalization of enterprises (DGL) has a mean value of 0.0875, a median value of 0.0077, and a standard deviation of 0.2185. This indicates that the degree of digital transformation among Chinese businesses is not uniform. Overall, the distribution of the remaining control variables appears relatively stable. Only the standard deviation of CURRENT RATIO is larger, indicating some variation in the cash flow profiles of Chinese companies, which is consistent with Shi’s findings (2019) [43].

**Table 3:** Descriptive statistic.

Variable	N	SD	Mean	Min	p50	Max
EP	31,315	0.0820	0.6373	0.3539	0.6666	0.7182
DGL	31,315	0.2185	0.0875	0.0000	0.0077	1.0000
lev	31,315	0.2228	0.4402	0.0490	0.4287	1.0590
cash	31,315	0.0752	0.0424	-0.2040	0.0429	0.2489
cur	31,315	2.7893	2.4866	0.2189	1.6067	18.0875
growth	31,315	0.8146	0.2746	-0.6988	0.1125	6.0512
tan	31,315	0.0898	0.9279	0.5133	0.9579	1.0000
ind	31,315	0.0534	0.3736	0.3077	0.3333	0.5714
isp	31,315	0.2454	0.4512	0.0032	0.4713	0.9375

### 4.2 Correlation analysis

Table 4 shows the results of the correlation analysis of the paper’s main variables. It shows that the degree of digitalization (DGL) has a significant positive relationship with the company’s environmental protection (EP). We have also conducted variance inflation factor test. Since the variance inflation factor is less than 10, we can infer that there is no co-linearity between the primary variables.

**Table 4:** Correlation analysis.

	EP	DGL	lev	cash	cur	growth	tan	ind	isp
EP	1								
DGL	0.117***	1							
lev	-0.082***	-0.026***	1						
cash	0.135***	0.052***	-0.157***	1					
cur	0.133***	0.092***	-0.624***	0.015***	1				
growth	0.071***	0.072***	0.033***	0.035***	-0.002	1			
tan	-0.014**	0.050***	0.070***	0.045***	0.108***	-0.038***	1		
ind	0.007*	0.032***	-0.008	-0.025***	0.020***	0.009*	-0.011**	1	
isp	-0.177***	-0.054***	0.194***	0.115***	-0.167***	0.023***	0.065***	-0.069***	1

Standard errors in brackets.

* p<0.1

** p<0.05

*** p<0.01.

### 4.3 Benchmark regression

Table 5 provides an empirical examination of the standard regression. Multiple regressions were used to control for industry fixed effects and time fixed effects in column (1) of Table 5. Evidently, a firm’s degree of digital transformation (DGL) has a significant impact on its environmental protection (EP) (0.0185 coefficient, significant at the 1% level). Considering that omitted variables may have an effect on the empirical results, we include a number of control variables that reflect the financial status and management characteristics of the businesses. In column (2) of Table 5, the empirical results are displayed. After the inclusion of the control variables, the coefficient decreases from 0.0185 to 0.0155, presumably because some of the factors influencing the environmental protection (EP) of the enterprise are absorbed. However, the significance did not change, as both were significant at the 1% level. The hypothesis of this investigation appears to be initially supported. Thus, the degree of digitalization (DGL) of a company can contribute considerably to its environmental protection (EP).

**Table 5:** Benchmark regression.

	(1)	(2)
	EP	EP
DGL	0.0185***	0.0155***
	[0.0031]	[0.0030]
controls	no	yes
industry	yes	yes
year	yes	yes
_cons	0.6357***	0.7077***
	[0.0011]	[0.0100]
N	31,315	31,315
adj. R-sq	0.2377	0.2844

Standard errors in brackets.

* p<0.1

** p<0.05

*** p<0.01.

### 4.4 Robustness tests

Multiple regressions confirmed in the preceding section that the degree of digitalization of the firm (DGL) can considerably contribute to the firm’s environmental protection (EP). This study will conduct a robustness test with replacement explanatory variables and a GMM model utilizing the 2SLS method.

This study executes robustness tests by varying the method of measuring explanatory variables. The test results are displayed in column (1) of Table 6. The effect of the degree of digitalization of firms (DGLs) on the environmental protection of firms (EP) remains substantially positive (coefficient of 1.151, significant at the 5% level) despite a change in the way explanatory variables are measured. At the 1% significance level, the F test for first-stage regression was 29.128.43 over 10, and the p-value was significant. The LM test result was 12.1, which is greater than 10, and the 1% significance level p-value is significant. In this investigation, additional instrumental variables were used to test for endogeneity. Through knowledge diffusion effects and competitive pressures, the degree of digitalization of firms in the same region and industry influences the degree of digitalization of the target firm [44], but has little direct effect on the environmental management of the firm. As a result, the target firm’s digitization mean in the same region and industry as the target firm is calculated and used as an instrumental variable. The results are shown in column 3 of Table 6, where the regional infrastructure has an effect on the digital transformation of the firm [45] but has no direct effect on the firm’s environmental protection as measured by the weak identification test and the underidentification test. Today’s digital technology is primarily carried by computers and other devices, and a robust Internet infrastructure can facilitate the digital transformation of businesses. As a result, the number of residences with broadband Internet access in the enterprise’s location serves as an instrumental variable in this study. Please refer to column 4 of Table 6 for the empirical findings, organized by the weak identification test and the underidentification test.

**Table 6:** Robustness test.

		(1)	(2)	(3)	(4)
		EP	EP	EP	EP
DGLs		1.151**	-	-	-
		[0.4541]	-	-	-
DGL		-	0.0175***	0.0206***	0.3313**
		-	[0.0029]	[0.0032]	[0.1485]
underidentification test	LM test	-	12.10	13,000.00	10.32
	P	-	0.00	0.00	0.00
weak identification test	F test in first-stage regressions	-	29,128.43	21,035.90	10.30
	P	-	0.00	0.00	0.00
controls		yes	yes	yes	yes
industry		yes	yes	yes	yes
year		yes	yes	yes	yes
_cons		0.6936***	0.7862***	0.7891***	0.8339***
		[0.0116]	[0.0076]	[0.0077]	[0.0247]
N		31,315	31,315	31,315	31,315
adj. R-sq		0.2833	0.2844	0.2843	0.2738

Standard errors in brackets.

* p<0.1

** p<0.05

*** p<0.01.

### 4.5 Further research

#### 4.5.1 Mesomeric effects

Businesses can get out of their financial binds and improve their financial situation through digital transformation, which gives them enough money for environmental management. Protecting the environment is important for the long-term growth of human society and for each person’s well-being. Companies have a moral duty to do their part to protect the environment as a social environment. Protection of the environment, on the other hand, is a way for businesses to internalize external diseconomies into the cost of doing business [46]. As an economic entity, a business exists to generate profit. Therefore, it is unreasonable to expect businesses to invest a disproportionate amount of their limited capital in environmental protection, regardless of their own business growth. This concept is implausible. Only when a business’s capital is healthy can it be concerned with environmental protection [47]. This research will analyze and empirically investigate the extent of firms’ external financing constraints and internal financial position. From the standpoint of funding constraints. External financing constraints are alleviated by the fact that the transformation of businesses is consistent with the policy direction of constructing "Digital China" and "Smart China." Enterprises that comply with the national policy orientation are more likely to receive government support in certain areas and to avoid certain hazards; consequently, capital market investors favor them [48]. This is exacerbated by the policy-oriented nature of China’s unique market economy. When a company is actively digitizing, the capital market tends to favor the company. As a consequence, its market value increases and its cost of capital employed decreases. Thus, it appears that digitalization can facilitate environmental protection behavior by reducing the extent of external financing restrictions. Analysis from the perspective of the financial situation of the company. The digitalization of businesses is not only an act of policy orientation but also the best course of action for businesses seeking to increase productivity [49]. The digitalization of businesses can provide numerous benefits to the businesses themselves [50]. In the context of digitalization, businesses have access to more accurate and timely information, enabling them to make more accurate and rational decisions [51]. In this context, the financial position of businesses improves, allowing them to invest in environmentally friendly initiatives.

Using stepwise regression, this study looks at how the role of external financing constraints and internal financial position can act as a mediator. This paper refers to Hadlock and Pierce (2010) to look at how external financing constraints act as a mediator [52]. Hadlock and Pierce use the SA index to measure the level of financing constraints that firms face. This index is a bad sign. If it has a high value, it means that the company has a harder time getting money. The coefficient of -0.2875, which is significant at the 1% level, shows that the digitalization of firms (DGL) greatly reduces the financing constraint (SA) that firms face (column (2) of Table 7). The relationship between the digitalization of firms (DGL) and environmental protection is partially mediated by the degree of financing constraint (SA), as shown in column 3 of Table 7. (EP). This paper utilizes Altman’s (1968) research and the Zscore index to measure the financial position of a company [53]. The Zscore index is a positive indicator, with a higher value indicating a stronger financial position. In columns (4) and (5) of Table 7, their empirical findings are analyzed. Digitalization (DGL) can contribute to environmental protection (EP) by enhancing a company’s financial position, as shown in the table (Zscore). To assure the validity of the empirical findings, a bootstrap test (1,000 samples) was performed on these two mediating effects. In addition, the results demonstrate that the degree of external financing constraints (SA) and internal financial position (Zscore) serve as mediators.

**Table 7:** Mesomeric effects.

	(1)	(2)	(3)	(4)	(5)
	EP	SA	EP	Zscore	EP
DGL	0.0155***	-0.2875***	0.0097***	0.2424**	0.0152***
	[0.0030]	[0.0745]	[0.0027]	[0.0122]	[0.0030]
SA			-0.0200***		
			[0.0007]		
Zscore					0.0009***
					[0.0001]
controls	yes	yes	yes	yes	yes
industry	yes	yes	yes	yes	yes
year	yes	yes	yes	yes	yes
_cons	0.7077***	2.4829***	0.7574***	4.3463***	0.7038***
	[0.0100]	[0.2238]	[0.0092]	[0.5816]	[0.0100]
N	31,315	31,315	31,315	31,315	31,315
adj. R-sq	0.2844	0.3809	0.3724	0.4976	0.2863

Standard errors in brackets.

* p<0.1

** p<0.05

*** p<0.01.

#### 4.5.2 Moderating effects

In the last section, we looked at how a company’s level of digitalization (DGL) affects environmental protection (EP) by way of the ways it sends information. What external factors can affect the relationship between the two parties? As the aggregate of the external environment in which firms transact, the market business environment influences all aspects of a firm’s initiation, production, and transactions [54]. Li Keqiang, former Premier of the State Council, signed State Council Decree No. 722 in 2019, which announced the Regulations on Optimizing the Business Environment. The Regulations on Optimizing the Business Environment define the market’s business environment as the external conditions, such as institutional factors, that enterprises in a market economy must contend with. The National Economic Research Institute (NERI) has devised the "Marketability Index," a positive indicator that considers government-business relations, market integrity, and finance. A higher value indicates a more favorable market environment. When the market environment improves, government regulations become more comprehensive. Streamlining government processes can increase business efficacy, decrease the costs firms incur when communicating with the government, and reduce the burden on businesses by reducing political rent-seeking [55]. Moreover, in a stable and regulated business environment, the capital market can serve as a "reservoir" more effectively, thereby alleviating companies’ capital access issues to some extent [56]. Thus, when the business environment is favorable, external uncertainty is reduced and firms spend less on maintaining the external environment’s stability, which is conducive to the allocation of scarce resources to long-term initiatives that benefit the environment. Table 8 column 2 displays the moderating effect of adding the effect of the business environment (markt). The effect of the digitalization of firms (DGL) on environmental protection increases when the business environment is included (DGL*markt, coefficient 0.0026, significant at the 10% level).

**Table 8:** Moderating effects.

	(1)	(2)	(3)
	EP	EP	EP
DGL	0.0155***	0.009*	0.0178***
	[0.0030]	[0.0176]	[0.0036]
DGL*markt		0.0026*	
		[0.0018]	
markt		0.0016**	
		[0.0007]	
DGL*HHI			-0.015*
			[0.0105]
HHI			-0.0063*
			[0.0043]
controls	yes	yes	yes
industry	yes	yes	yes
year	yes	yes	yes
_cons	0.7077***	0.7231***	0.7085***
	[0.0100]	[0.0116]	[0.0100]
N	31,315	31,315	31,315
adj. R-sq	0.2844	0.2851	0.2845

Standard errors in brackets.

* p<0.1

** p<0.05

*** p<0.01.

The degree of competition in an industry refers to the intensity of competition between enterprises in the industry at a particular time. In industries with greater levels of competition, firms frequently encounter a plethora of rivals with the same business strategy. To make sure their products sell well, these companies often compete with each other in a zero-sum way, such as by lowering their prices to take the market [57]. When the situation gets bad enough, the company’s operating costs will go up, and as a result, the company’s income may go down (Chen et al., 2021). So, at this point in time, the company may face more operational risk, and its financial situation is getting worse [58]. In this situation, we can’t expect businesses that are focused on making money to care too much about protecting the environment. The Herfindahl-Hirschman Index is used in this study to measure how competitive an industry is. A higher index means that the industry is more competitive. In column 3 of Table 8, the moderating effect of the intervention on the degree of market competition is examined (HHI). When there is a lot of competition in the market, digitalization of firms (DGL) has less of a positive effect on environmental protection (EP). The coefficient of DGL*HHI is -0.015, which is significant at the 10% level.

#### 4.5.3 Threshold effect

When it comes to outside monitoring of businesses, a high level of investor confidence is not good for the smooth running of businesses. Behavioral finance theory says that investors don’t act in a logical way and that their mental states show up in how they act [59]. In other words, when investors’ emotions are elevated, their behavior becomes so aggressive that they neglect to monitor the company [60], which may exacerbate the first type of agency problems. Under the modern corporate system, in which ownership and operation of the business are separated, shareholders may employ managers with certain competencies to assist them in managing the business if they lack the necessary time and/or resources. The manager may not have a healthy mental state because he or she has to put a lot of work into the business but only gets a small share of the profits. This, along with the fact that managers have greater access to and influence over the actual production and operation of the business, is likely to result in "self-interested" decisions regarding the actual production and operation of the business [61]. In addition to encouraging firms to engage in more transactions with their own favored parties, managers frequently engage in self-serving behavior by raising "agency costs" [62]. In the absence of external oversight, the firm’s agency costs increase further, putting at risk the funds available to enhance the quality of its development [63]. At this juncture, the firm’s financial security may be at risk. In the absence of external oversight, a company is also susceptible to a tumultuous and ineffective business environment. The current decisions made by businesses may not be forward-looking (To et al., 2018) [64]. Then, companies may not value initiatives with long-term benefits, such as digitalization and environmental protection. And, as investor sentiment improves, the absence of external corporate supervision may have an impact on the form that corporate digitalization for environmental protection can take. Digitalization provides stakeholders with additional information. Nonetheless, excessive information may have a negative impact on company management [65]. An excessive amount of accounting information at this time becomes irrelevant to the managerial decision-making process and disrupts the regulation of external stakeholders. The new business model brought about by digitalization allows managers to engage in "selfish" behavior during periods of high investor sentiment. We contend that when investor sentiment exceeds a certain threshold, digitalization can have a negative effect on environmental protection.

In this paper, we use Baker & Wurgler’s (2007) method of decomposing the Tobinq value to measure investor sentiment [66]. Table 9 displays the threshold value test, where an investor sentiment threshold value is determined (threshold value test: -6.3765, p-value: 0.0830). Table 10 shows the results of the threshold regression. Before the threshold value is reached, it is evident that the digitalization of companies (DGL) has a positive impact on environmental protection (EP). However, once the threshold is exceeded, DGL has a detrimental effect on EP.

**Table 9:** Threshold value test.

	Threshold	RSS	MSE	Fstat	Prob	Crit10	Crit5	Crit1
Single	-6.3765	2.9733	0.0001	59.8100	0.0830	55.5600	66.1230	89.3600
Double	-5.5312	2.9738	0.0001	-5.1400	1.0000	74.9360	94.4600	143.1660

**Table 10:** Threshold regression.

	(1)	(2)
	EP	EP
	IC<-6.3765	IC>-6.3765
DGL	0.0072*	-0.0140***
	[0.0040]	[0.0023]
controls	yes	
_cons	0.5837***	
	[0.0062]	
N	31,315	
adj. R-sq	-0.1133	

Standard errors in brackets.

* p<0.1

** p<0.05

*** p<0.01.

## 5 Discussion and recommendation

The digitalization of businesses can contribute to environmental preservation by improving the financial situation and reducing the level of financing restrictions. When digitalization is used in businesses, this advanced technology can be used to cut costs, increase efficiency, and improve production methods [67]. This protects the environment by increasing output per unit and decreasing energy consumption. However, we must also consider the potential negative consequences of digitalization [68]. Despite the fact that digitalization provides companies and their stakeholders with a number of benefits, the vast quantities of information it accumulates constitute a form of noise. If the communication platforms created by digital information technology are not properly planned, the benefits of digitalization, such as quick communication, may have a negative impact on the company’s normal operations [69].

The government must foster the development of the digital economy and provide mental guidance. The growth of the digital economy can help human society integrate resources more effectively and establish a more rational communication platform. In addition, prior to constructing a digital platform, the government should perform an effective role of external oversight to ensure that the platform is fundamentally sound. Additionally, government policies and regulations can affect the preferences of the capital market. China’s government can play an effective role as the "invisible hand" by urging businesses to protect the environment in order to guarantee their own survival and growth. The dangers of over-concentration should also be brought to the attention of the government. Too much concentration can lead to limited resources being held in the hands of a few, making it impossible to maximize the use of resources. When bullish, investors must also "throw frigid water" on the market. High investor sentiment can be advantageous for companies’ capital costs, but the resulting absence of regulation can prohibit the use of digitalization-generated resources for environmental protection.

## 6 Conclusion, limitations, and future research scope

### 6.1 Conclusion

This study uses text analysis and the Entropy-TOPSIS method to come up with an indicator for judging how well companies take care of the environment. After doing the necessary work on the data, we got 31,315 data samples and came to the following conclusions: (1) Enterprise digitalization contributes to environmental protection. As digitalization expands, the production and management practices of businesses are altered. With the implementation of modern information technology, it is anticipated that enterprise output per unit will increase substantially, while energy consumption will decrease. This will assist businesses in protecting the environment. (2) The market environment and level of industry competition perform a moderating role. In a healthy business environment, production, transactions, and distribution in the marketplace typically flow smoothly and orderly. The environmental repercussions of digitalization are effectively communicated, and access to financing is ensured. The increased market competition will inevitably have an effect on the day-to-day operations of the business. In a highly competitive market, businesses must concentrate on their survival (competition). In this instance, digitalization’s resource and management advantages are prioritized for product competition. Not immediately profitable projects, such as environmental protection, are frequently neglected. (3) Digitalization is not perfect for businesses. When investor sentiment is so high that external oversight is compromised. The convenience of digitalization and the vast quantity of available information can serve as a cover for managers’ "self-interested" behavior. It can even interfere with business decisions.

### 6.2 Limitations and future research scope

This study’s sample selection has some limitations. For the sake of data consistency, we use the Chinese Shanghai and Shenzhen A-shares from 2008 to 2019 as the sample, omitting data from the post-COVID-19 period. Taking into account the period requirement of the research methodology and the schedule of financial reports issued by Chinese companies that are publicly traded. After the listed companies have produced their corporate financial statements for 2022, academics will be able to examine the effect of corporate digitalization on environmental protection in terms of COVID-19.

## Supporting information

S1 Fig (DOCX)

S1 Data (DTA)

## References

[1] Chen J., & Zhao D. (2022). Complexity of domestic production fragmentation and its impact on pollution emissions: Evidence from decomposed regional production length. Structural Change and Economic Dynamics, 61, 127–137.

[2] Babamiri O., Vanaei A., Guo X., Wu P., Richter A., & Ng K. T. W. (2021). Numerical Simulation of Water Quality and Self-Purification in a Mountainous River Using QUAL2KW. Journal of Environmental Informatics, 37(1).

[3] Peng H., Shen N., Ying H., & Wang Q. (2021). Can environmental regulation directly promote green innovation behavior?——based on situation of industrial agglomeration. Journal of Cleaner Production, 314, 128044.

[4] Fan W., Wang S., Gu X., Zhou Z., Zhao Y., & Huo W. (2021). Evolutionary game analysis on industrial pollution control of local government in China. Journal of Environmental Management, 298, 113499. doi: 10.1016/j.jenvman.2021.113499 34385115

[5] Chen Y., Xu S., Lyulyov O., & Pimonenko T. (2022). China’s digital economy development: incentives and challenges. Technological and Economic Development of Economy, 1–21.

[6] Jordan T., & Richterich A. (2022). Researching the digital economy and the creative economy: Free gaming shards and commercialised making at the intersection of digitality and creativity. European Journal of Cultural Studies, 13675494221118390.

[7] Shahbaz M., Wang J., Dong K., & Zhao J. (2022). The impact of digital economy on energy transition across the globe: The mediating role of government governance. Renewable and Sustainable Energy Reviews, 166, 112620.

[8] Cui X., Wang P., Sensoy A., Nguyen D. K., & Pan Y. (2022). Green credit policy and corporate productivity: evidence from a quasi-natural experiment in China. Technological Forecasting and Social Change, 177, 121516.

[9] Hoque A., & Clarke A. (2013). Greening of industries in Bangladesh: pollution prevention practices. Journal of Cleaner Production, 51, 47–56.

[10] Shen Y., & Zhang X. (2022). Study on the Impact of Environmental Tax on Industrial Green Transformation. International Journal of Environmental Research and Public Health, 19(24), 16749. doi: 10.3390/ijerph192416749 36554630 PMC9779415

[11] Halbert L., Henneberry J., & Mouzakis F. (2014). Finance, business property and urban and regional development. Regional Studies, 48(3), 421–424.

[12] Sievers M., & Vandenberg P. (2007). Synergies through linkages: Who benefits from linking micro-finance and business development services?. World development, 35(8), 1341–1358.

[13] Xiang D., Zhang Y., & Worthington A. C. (2018). Determinants of the use of fintech finance among Chinese small and medium-sized enterprises. In 2018 IEEE international symposium on innovation and entrepreneurship (TEMS-ISIE) (pp. 1–10). IEEE.

[14] Dou J., & Han X. (2019). How does the industry mobility affect pollution industry transfer in China: Empirical test on Pollution Haven Hypothesis and Porter Hypothesis. Journal of cleaner production, 217, 105–115.

[15] Li C., Xia W., & Wang L. (2021). The transfer mechanism of pollution industry in China under multi-factor combination model—Based on the perspective of industry, location, and environment. Environmental Science and Pollution Research, 28, 60167–60181. doi: 10.1007/s11356-021-14643-6 34151403

[16] Chen J., Chen K., Wang G., Wu L., Liu X., & Wei G. (2019). PM2. 5 pollution and inhibitory effects on industry development: A bidirectional correlation effect mechanism. International journal of environmental research and public health, 16(7), 1159. doi: 10.3390/ijerph16071159 30935121 PMC6480563

[17] Zhao L., & Chen L. (2022). Research on the impact of government environmental information disclosure on green total factor productivity: empirical experience from Chinese province. International Journal of Environmental Research and Public Health, 19(2), 729. doi: 10.3390/ijerph19020729 35055551 PMC8775407

[18] Zhang Y., Li X., Song Y., & Jiang F. (2021). Can green industrial policy improve total factor productivity? Firm-level evidence from China. Structural Change and Economic Dynamics, 59, 51–62.

[19] Liu Y., Liu C., & Zhou M. (2021). Does digital inclusive finance promote agricultural production for rural households in China? Research based on the Chinese family database (CFD). China Agricultural Economic Review, 13(2), 475–494.

[20] Wang P., Han W., Rizvi S. K. A., & Naqvi B. (2022). Is digital adoption the way forward to curb energy poverty?. Technological Forecasting and Social Change, 180, 121722.

[21] Wu B., & Yang W. (2022). Empirical test of the impact of the digital economy on China’s employment structure. Finance Research Letters, 49, 103047.

[22] Rief S. (2023). Karl Polanyi’s ‘socialist accounting’and ‘overview’in the age of data analytics. New Political Economy, 28(2), 284–298.

[23] Rowbottom N., Locke J., & Troshani I. (2021). When the tail wags the dog? Digitalization and corporate reporting. Accounting, Organizations and Society, 92, 101226.

[24] Jin W., Zhang H. Q., Liu S. S., & Zhang H. B. (2019). Technological innovation, environmental regulation, and green total factor efficiency of industrial water resources. Journal of cleaner production, 211, 61–69.

[25] Lattacher W., Gregori P., Holzmann P., & Schwarz E. J. (2021). Knowledge spillover in entrepreneurial emergence: A learning perspective. Technological Forecasting and Social Change, 166, 120660.

[26] Zarte M., Pechmann A., & Nunes I. L. (2022). Knowledge framework for production planning and controlling considering sustainability aspects in smart factories. Journal of Cleaner Production, 363, 132283.

[27] Beaulieu M., & Bentahar O. (2021). Digitalization of the healthcare supply chain: A roadmap to generate benefits and effectively support healthcare delivery. Technological forecasting and social change, 167, 120717.

[28] Jahn H. K., Jahn I. H. J., Behringer W., Lyttle M. D., Roland D., & Paediatric Emergency Research United Kingdom and Ireland (PERUKI). (2021). A survey of mHealth use from a physician perspective in paediatric emergency care in the UK and Ireland. European Journal of Pediatrics, 180, 2409–2418. 10.1007/s00431-021-04023-0 PMC8285308 33763717

[29] Del Giudice M., Scuotto V., Garcia-Perez A., & Petruzzelli A. M. (2019). Shifting Wealth II in Chinese economy. The effect of the horizontal technology spillover for SMEs for international growth. Technological Forecasting and Social Change, 145, 307–316.

[30] Karahanna E., Chen A., Liu Q. B., & Serrano C. (2019). Capitalizing on health information technology to enable digital advantage in US hospitals. MIS quarterly, 43(1), 113–140.

[31] Yao J., & Deng Z. (2016). Dynamic resource integration optimisation of global distributed manufacturing: an embeddedness–interaction perspective. International Journal of Production Research, 54(23), 7143–7157.

[32] Niu F., & Jiang Y. (2021). Economic sustainability of China’s growth from the perspective of its resource and environmental supply system: National scale modeling and policy analysis. Journal of Geographical Sciences, 31(8), 1171–1186.

[33] Pan Y., Zhang B., Wu Y., & Tian Y. (2021). Sustainability assessment of urban ecological-economic systems based on emergy analysis: A case study in Simao, China. Ecological Indicators, 121, 107157.

[34] Liu X., & Liu F. (2022). Environmental regulation and corporate financial asset allocation: A natural experiment from the new environmental protection law in China. Finance Research Letters, 47, 102974.

[35] Ma Y., & Li B. (2022). Effect of digitalization on knowledge transfer from universities to enterprises: Evidence from postdoctoral workstation of Chinese enterprises. Technology in Society, 71, 102102.

[36] Huang Z., Kim J., Sadri A., Dowey S., & Dargusch M. S. (2019). Industry 4.0: Development of a multi-agent system for dynamic value stream mapping in SMEs. Journal of Manufacturing Systems, 52, 1–12.

[37] Byun J., Kim K., Liao R. C., & Pan C. (2021). The impact of investor sentiment on catering incentives around the world. Journal of International Financial Markets, Institutions and Money, 71, 101285.

[38] Huang A. H., Zang A. Y., & Zheng R. (2014). Evidence on the information content of text in analyst reports. The Accounting Review, 89(6), 2151–2180.

[39] Feia Wu, Huizhib Hu, Huiyanc Lin & Xiaoyi Ren. (2021). Enterprise Digital Transformation and Capital Market Performance:Empirical Evidence from Stock Liquidity. Journal of Management World, (07),130–144+10. doi: 10.19744/j.cnki.11-1235/f.2021.0097 (in Chinese)

[40] Liang X., Luo L., Hu S., & Li Y. (2022). Mapping the knowledge frontiers and evolution of decision making based on agent-based modeling. Knowledge-Based Systems, 250, 108982.

[41] Forchtner B., & Özvatan Ö. (2022). De/legitimising EUrope through the performance of crises: The far-right Alternative for Germany on “climate hysteria” and “corona hysteria”. Journal of Language and Politics, 21(2), 208–232.

[42] Zhang P., Wu F., Guo Y., & Ma J. (2022). Does enforcement matter in promoting corporate environmental investment: Evidence from Chinese private firms. Journal of Cleaner Production, 337, 130432.

[43] Shi M. (2019). Overinvestment and corporate governance in energy listed companies: Evidence from China. Finance Research Letters, 30, 436–445.

[44] Tang C., Qiu P., & Dou J. (2022). The impact of borders and distance on knowledge spillovers—Evidence from cross-regional scientific and technological collaboration. Technology in Society, 70, 102014.

[45] Wang Z., He Q., Xia S., Sarpong D., Xiong A., & Maas G. (2020). Capacities of business incubator and regional innovation performance. Technological Forecasting and Social Change, 158, 120125.

[46] Freeling B. S., & Connell S. D. (2020). Funding conservation through an emerging social movement. Trends in Ecology & Evolution, 35(1), 3–6. doi: 10.1016/j.tree.2019.09.002 31615635

[47] Yang J., Shi D., & Yang W. (2022). Stringent environmental regulation and capital structure: The effect of NEPL on deleveraging the high polluting firms. International Review of Economics & Finance, 79, 643–656.

[48] Bouri E., Demirer R., Gabauer D., & Gupta R. (2022). Financial market connectedness: The role of investors’ happiness. Finance Research Letters, 44, 102075.

[49] Brewer S., Pearson S., Maull R., Godsiff P., Frey J. G., Zisman A., et al. (2021). A trust framework for digital food systems. Nature Food, 2(8), 543–545. doi: 10.1038/s43016-021-00346-1 37118166

[50] Trinugroho I., Pamungkas P., Wiwoho J., Damayanti S. M., & Pramono T. (2022). Adoption of digital technologies for micro and small business in Indonesia. Finance Research Letters, 45, 102156.

[51] Ademi P., Schuhmacher M. C., & Zacharakis A. L. (2022). Evaluating Affordance-Based Opportunities: A Conjoint Experiment of Corporate Venture Capital Managers’ Decision-Making. Entrepreneurship Theory and Practice, 10422587221134788.

[52] Hadlock C. J., & Pierce J. R. (2010). New evidence on measuring financial constraints: Moving beyond the KZ index. The review of financial studies, 23(5), 1909–1940.

[53] Altman E. I. (1968). Financial ratios, discriminant analysis and the prediction of corporate bankruptcy. The journal of finance, 23(4), 589–609.

[54] Boamah N. A. (2022). Segmentation, business environment and global informational efficiency of emerging financial markets. The Quarterly Review of Economics and Finance, 84, 52–60.

[55] Peco-Torres F., Polo-Peña A. I., & Frías-Jamilena D. M. (2021). Revenue management and CRM via online media: The effect of their simultaneous implementation on hospitality firm performance. Journal of Hospitality and Tourism Management, 47, 46–57.

[56] Liu Z., Li W., Hao C., & Liu H. (2021). Corporate environmental performance and financing constraints: An empirical study in the Chinese context. Corporate Social Responsibility and Environmental Management, 28(2), 616–629.

[57] He Q., Liu J., & Zhang C. (2021). Exchange rate exposure and international competition: Evidence from Chinese industries. Journal of Contemporary China, 30(131), 820–840.

[58] Chen T., Kenneth Cheng H., Jin Y., Li S., & Qiu L. (2021). Impact of competition on innovations of it industry: An empirical investigation. Journal of management information systems, 38(3), 647–666.

[59] Singh J E, Babshetti V, Shivaprasad H N. Efficient Market Hypothesis to Behavioral Finance: A Review of Rationality to Irrationality[J]. Materials Today: Proceedings, 2021(3).

[60] Dong H., & Gil-Bazo J. (2020). Sentiment stocks. International Review of Financial Analysis, 72, 101573.

[61] Newbert S. L. (2018). Achieving social and economic equality by unifying business and ethics: Adam Smith as the cause of and cure for the separation thesis. Journal of Management Studies, 55(3), 517–544.

[62] Bosse D. A., & Phillips R. A. (2016). Agency theory and bounded self-interest. Academy of management review, 41(2), 276–297.

[63] Trinh Q. D., Haddad C., & Tran K. T. (2022). Financial reporting quality and dividend policy: New evidence from an international level. International Review of Financial Analysis, 80, 102026.

[64] To T. Y., Navone M., & Wu E. (2018). Analyst coverage and the quality of corporate investment decisions. Journal of Corporate Finance, 51, 164–181.

[65] Salvi A., Vitolla F., Rubino M., Giakoumelou A., & Raimo N. (2021). Online information on digitalization processes and its impact on firm value. Journal of Business Research, 124, 437–444.

[66] Baker M., & Wurgler J. (2007). Investor sentiment in the stock market. Journal of economic perspectives, 21(2), 129–151.

[67] Falentina A. T., Resosudarmo B. P., Darmawan D., & Sulistyaningrum E. (2021). Digitalization and the performance of micro and small enterprises in Yogyakarta, Indonesia. Bulletin of Indonesian Economic Studies, 57(3), 343–369.

[68] Kortmann L. K., Simonson J., Vogel C., & Huxhold O. (2021). Digitalization and employees’ subjective job quality in the second half of working life in Germany. Social indicators research, 1–21. 10.1007/s11205-021-02854-w PMC8635314 34873360

[69] Kunkel S., Matthess M., Xue B., & Beier G. (2022). Industry 4.0 in sustainable supply chain collaboration: Insights from an interview study with international buying firms and Chinese suppliers in the electronics industry. Resources, conservation and recycling, 182, 106274.

